# Supplementary material for: Behavioural rhythms of two amphipod species Marinogammarus marinus and Gammarus pulex under increasing levels of light at night
Source: PLoS One. 2025 Aug 7;20(8):e0329449. doi: 10.1371/journal.pone.0329449 (PMC12331069; doi:10.1371/journal.pone.0329449)
Supplement: S1 Table — Design is the same as in Table 2 of main manuscript but separated for female (f) and male (m) M. marinus and G. pulex. (DOCX) [file pone.0329449.s001.docx]

**S1 Table Behavioural parameters of *E. marinus* and *G. pulex* activity under varying light/dark cycles.** Design is the same as in Table 2 of main manuscript but separated for female (f) and male (m) *E. marinus* and *G. pulex.*

| **Species** | **Light regime** | **n_tot_** | | **n_al_** | | **n_act_** | | **%_al_** | | **%_act_** | | **Rhythmicity** | | | | **Total activity counts** | | | | **Nocturnality** | | | | **Period (hours)** | | | |
| --- | --- | --- | --- | --- | --- | --- | --- | --- | --- | --- | --- | --- | --- | --- | --- | --- | --- | --- | --- | --- | --- | --- | --- | --- | --- | --- | --- |
|  |  | **f** | **m** | **f** | **m** | **f** | **m** | **f** | **m** | **f** | **m** | **Avg_f_** | **± SE** | **Avg_m_** | **± SE** | **Avg_f_** | **± SE** | **Avg_m_** | **± SE** | **Avg_f_** | **± SE** | **Avg_m_** | **± SE** | **Avg_f_** | **± SE** | **Avg_m_** | **± SE** |
| *E. marinus* | LD | 15 | 17 | 15 | 17 | 15 | 17 | 100.0 | 100.0 | 100.0 | 100.0 | 2.19 | 0.15 | 2.54 | 0.23 | 2590 | 318 | 2640 | 370 | 0.73 | 0.03 | 0.75 | 0.03 | 24.0 | 0.05 | 23.7 | 0.32 |
|  | LA01 | 16 | 16 | 15 | 15 | 15 | 14 | 100.0 | 93.8 | 100.0 | 93.3 | 1.64 | 0.29 | 1.36 | 0.10 | 1964 | 424 | 1206 | 191 | 0.43 | 0.04 | 0.44 | 0.05 | 23.5 | 0.50 | 24.9 | 0.37 |
|  | LA05 | 13 | 19 | 10 | 11 | 10 | 10 | 76.9 | 57.9 | 100.0 | 90.1 | 1.31 | 0.19 | 1.39 | 0.14 | 1091 | 595 | 618 | 87 | 0.47 | 0.05 | 0.52 | 0.05 | 24.8 | 0.31 | 23.1 | 0.97 |
|  | LA30 | 16 | 16 | 16 | 14 | 15 | 14 | 100 | 87.5 | 93.8 | 100.0 | 1.36 | 0.20 | 1.36 | 0.12 | 2293 | 1244 | 730 | 115 | 0.43 | 0.04 | 0.45 | 0.03 | 23.9 | 0.33 | 23.9 | 0.07 |
|  | LA50 | 16 | 16 | 13 | 13 | 12 | 12 | 81.3 | 81.3 | 92.3 | 92.3 | 0.86 | 0.10 | 0.99 | 0.16 | 884 | 105 | 1195 | 587 | 0.43 | 0.03 | 0.46 | 0.05 | 26.4 | 2.06 | 24.3 | 0.14 |
|  | LA80 | 17 | 15 | 15* | 8* | 14 | 8 | 88.2 | 53.3 | 93.3 | 100.0 | 1.49 | 0.10 | 1.75 | 0.25 | 736 | 108 | 869 | 271 | 0.66 | 0.04 | 0.63 | 0.09 | 23..5 | 0.35 | 25.5 | 1.25 |
|  | LL | 16 | 16 | 16 | 15 | 8* | 14* | 100 | 93.8 | 50.0 | 93.3 | 0.99** | 0.08 | 1.72** | 0.23 | 424*** | 117 | 1548*** | 414 | 0.63 | 0.06 | 0.68 | 0.04 | 29.3 | 2.77 | 23.4 | 0.56 |
|  | DD | 32 | 32 | 16 | 18 | 14 | 14 | 50.0 | 56.3 | 87.5 | 77.8 | 0.90 | 0.07 | 0.96 | 0.04 | 1206 | 667 | 896 | 377 | 0.49 | 0.06 | 0.46 | 0.04 | 24.9 | 1.30 | 24.0 | 1.04 |
| *G. pulex* | LD | 16 | 16 | 15 | 15 | 8* | 14* | 93.8 | 93.8 | 53.3 | 93.3 | 1.16 | 0.13 | 1.32 | 0.18 | 945 | 314 | 1442 | 231 | 0.46 | 0.09 | 0.59 | 0.04 | 25.0 | 1.01 | 25.4 | 1.59 |
|  | LA01 | 16 | 16 | 15 | 15 | 12 | 15 | 93.8 | 93.8 | 80.0 | 100.0 | 0.79 | 0.08 | 0.95 | 0.06 | 1229** | 281 | 2567** | 487 | 0.45 | 0.04 | 0.53 | 0.03 | 24.5 | 4.77 | 24.5 | 0.55 |
|  | LA05 | 16 | 16 | 13 | 16 | 11 | 16 | 81.3 | 100.0 | 84.6 | 100.0 | 1.00 | 0.10 | 1.02 | 0.10 | 1712 | 499 | 2156 | 400 | 0.48 | 0.05 | 0.49 | 0.04 | 25.4 | 1.63 | 26.4 | 1.24 |
|  | LA30 | 17 | 15 | 17 | 15 | 9* | 13* | 100.0 | 100.0 | 52.9 | 86.7 | 0.94** | 0.16 | 1.46** | 0.14 | 673 | 157 | 1492 | 522 | 0.48 | 0.07 | 0.45 | 0.05 | 29.5 | 5.50 | 24.8 | 1.43 |
|  | LA50 | 16 | 16 | 13 | 13 | 8 | 13 | 81.3 | 81.3 | 61.5 | 100.0 | 0.83 | 0.13 | 0.97 | 0.06 | 907 | 263 | 846 | 151 | 0.52 | 0.06 | 0.47 | 0.04 | 23.5 | 2.18 | 26.4 | 1.32 |
|  | LA80 | 16 | 16 | 12 | 15 | 10 | 13 | 75.0 | 93.8 | 83.3 | 86.7 | 0.96 | 0.14 | 1.24 | 0.14 | 746 | 116 | 1501 | 326 | 0.43 | 0.05 | 0.36 | 0.04 | 24.2 | 0.44 | 24.6 | 0.39 |
|  | LL | 16 | 15 | 15 | 13 | 12 | 10 | 93.8 | 86.7 | 80.0 | 76.9 | 0.89 | 0.06 | 0.86 | 0.12 | 1010 | 157 | 1468 | 431 | 0.56 | 0.05 | 0.53 | 0.04 | 23.3 | 2.68 | 27.0 | 2.33 |
|  | DD | 16 | 16 | 16 | 15 | 15 | 15 | 100 | 93.8 | 93.8 | 100.0 | 0.79 | 0.06 | 0.79 | 0.05 | 1640* | 420 | 2892* | 1894 | 0.46 | 0.02 | 0.48 | 0.01 | 25.0 | 1.00 | 24.8 | 3.25 |
| ntot = total number of individuals at the beginning of the assays; nal = # of individuals alive at the end of the eight day assays; %al = (nal /ntot)·100; nact = # of active individuals with total counts ≥ 200; %act = (nact/nal)·100; Avg = average; SE = standard error; %SR = % active individuals showing strongly rhythmic behaviour (RRP ≥ 1.5); %WR = % active individuals showing weakly rhythmic behaviour (RRP = 1–1.49); %AR = % active individuals showing arhythmic behaviour (RRP < 1). Different superscript letters denote significant differences (p < 0.05) between treatments from the Fisher’s exact tests and the Mann-Whitney U-tests. *E. marinus* and *G. pulex* were analysed separately. | | | | | | | | | | | | | | | | | | | | | | | | | | | |
