# Supplementary figures and images for: Behavioural rhythms of two amphipod species Marinogammarus marinus and Gammarus pulex under increasing levels of light at night
Source: PLoS One. 2025 Aug 7;20(8):e0329449. doi: 10.1371/journal.pone.0329449 (PMC12331069; doi:10.1371/journal.pone.0329449)

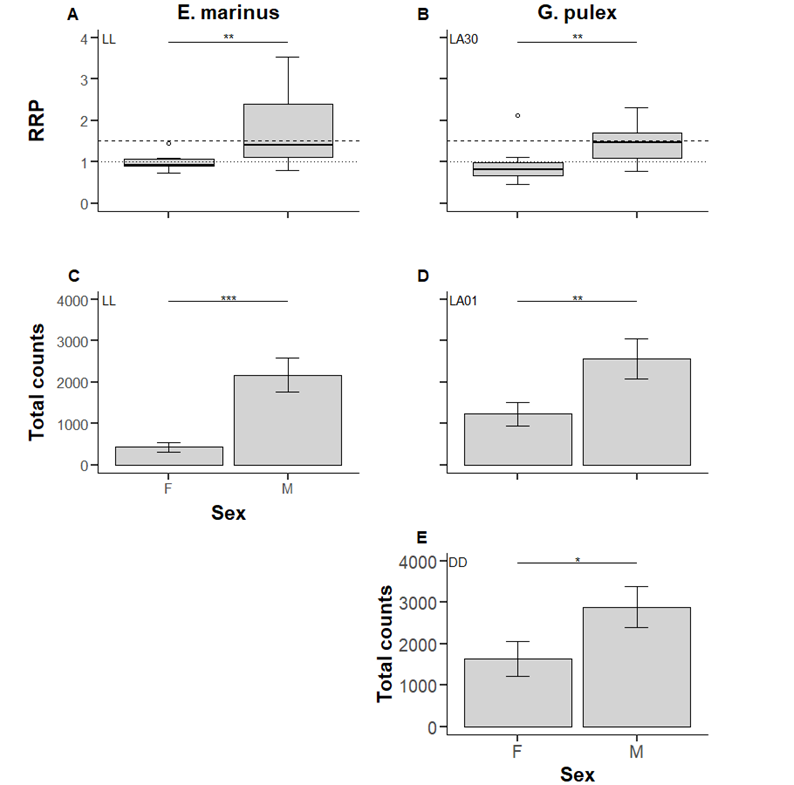

Supplement: S1 Fig — Boxplots of relative rhythmic power (RRP; A-B) – how strongly the activity repeated over a set period (e.g., 24 hours). Dotted line = 1 (rhythmic); Dashed line = 1.5 (strongly rhythmic). Barplots of total counts (C-E) – total amount of activity counts logged during the seven-day assay period. Assays occurred over seven days with 3-hour ramping between light transitions. Asterisks denote significance levels from the Mann-Whitney U-tests (0.5*0.1**0.001***0.0001). Light conditions outlined in Table 1 of main manuscript. (TIF) [file pone.0329449.s002.tif]

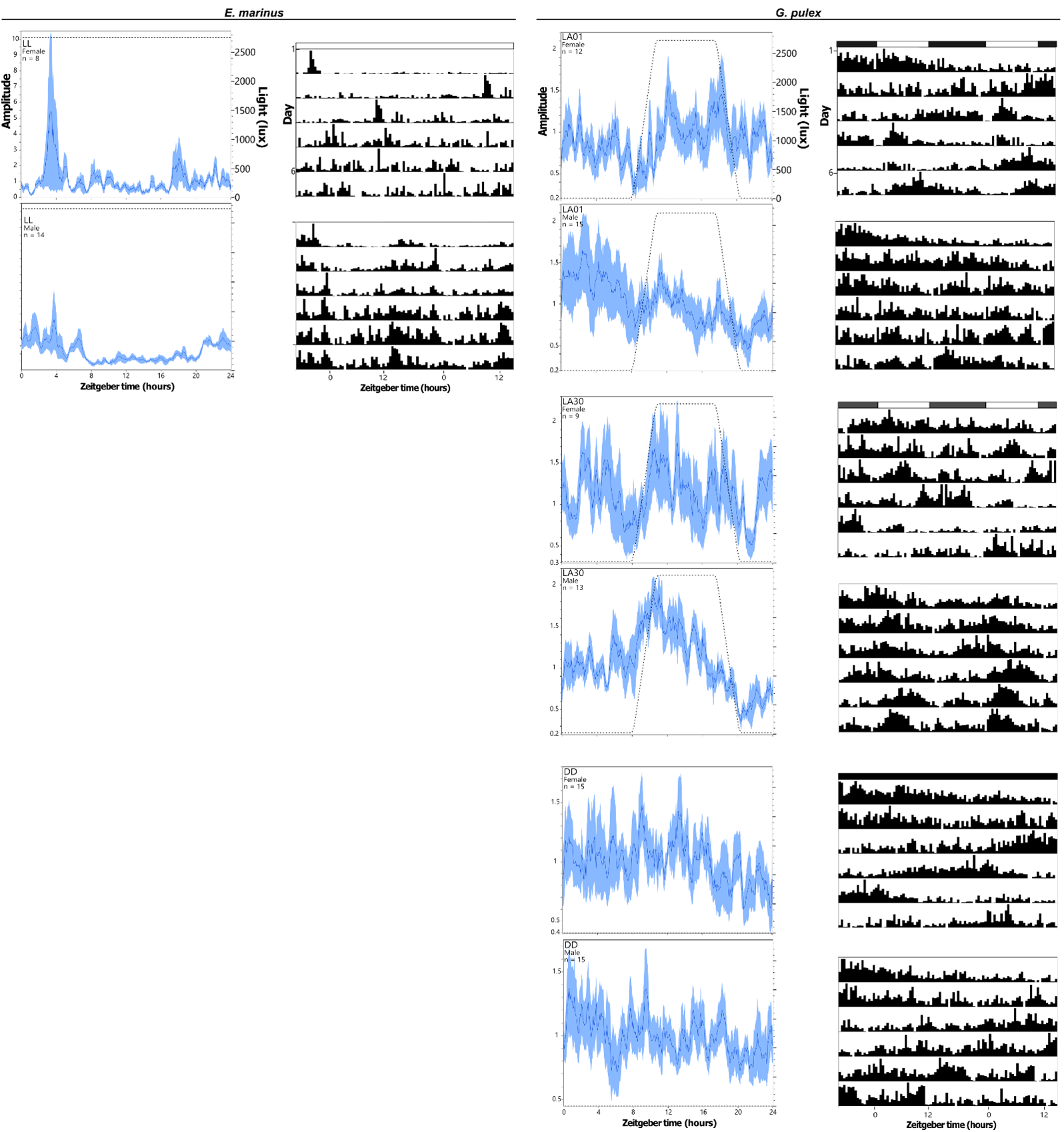

Supplement: S2 Fig — Activity profiles display average activity levels (black line) with standard deviation (grey areas), along with the light levels (dotted lines) over 24 hours. Actograms display the average, normalised behaviour across the 7-day assays, with each row showing two 24-hour cycles. Bars above actograms denote light levels; on/off transitions are not shown. Y axes are the same within columns. Light conditions outlined in Table 1 of main manuscript. (TIF) [file pone.0329449.s003.tif]

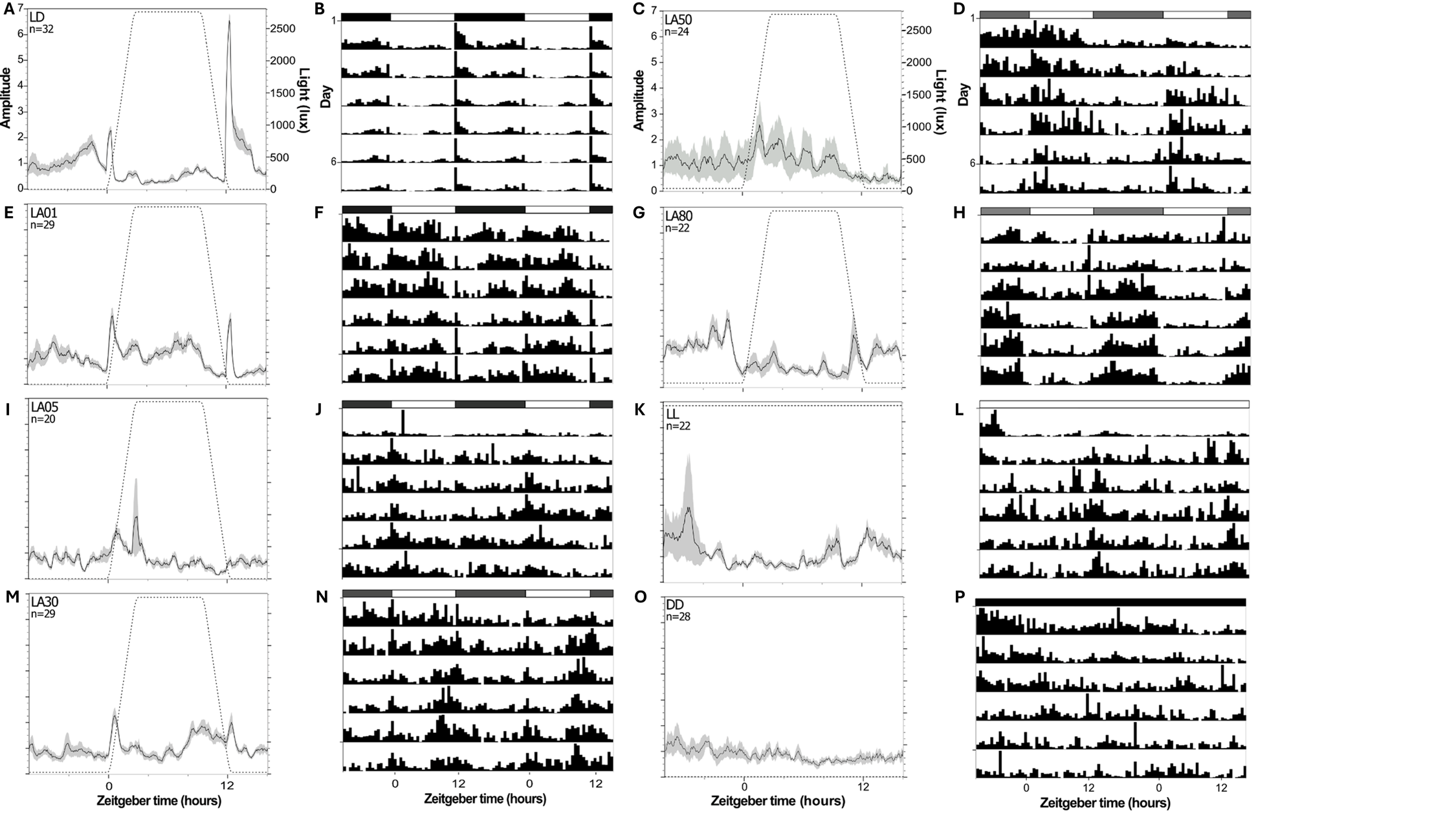

Supplement: S3 Fig — Activity profiles display average activity levels (black line) with standard deviation (grey areas), along with the light levels (dotted lines) over 24 hours. Actograms display the average, normalised behaviour across the 7-day assays, with each row showing two 24-hour cycles. Bars above actograms denote light levels; on/off transitions are not shown. Y axes are the same within columns. Light conditions outlined in Table 1 of main manuscript. (TIF) [file pone.0329449.s004.tif]

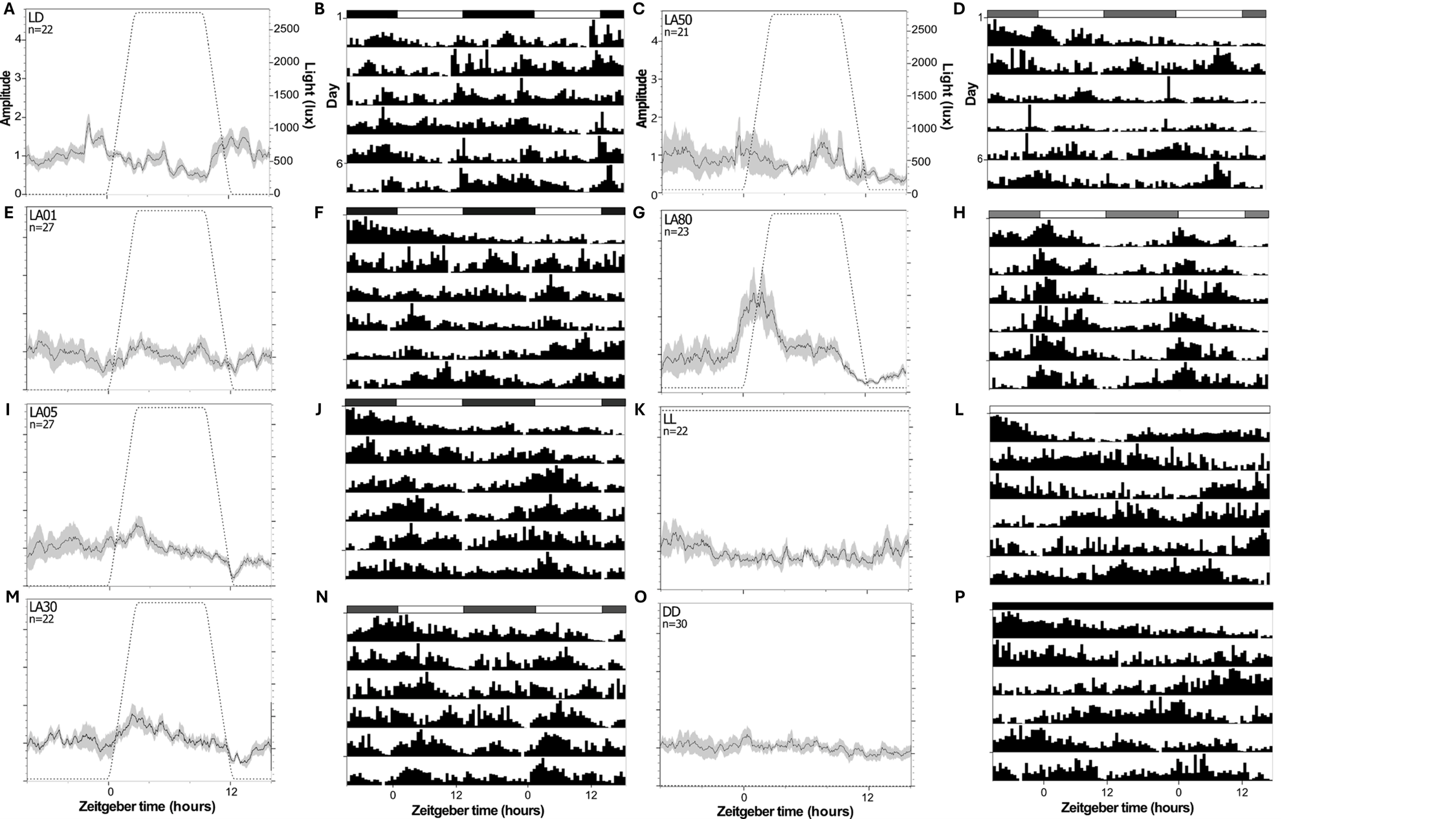

Supplement: S4 Fig — Activity profiles display average activity levels (black line) with standard deviation (grey areas), along with the light levels (dotted lines) over 24 hours. Actograms display the average, normalised behaviour across the 7-day assays, with each row showing two 24-hour cycles. Bars above actograms denote light levels; on/off transitions are not shown. Y axes are the same within columns. Light conditions outlined in Table 1 of main manuscript. (TIF) [file pone.0329449.s005.tif]
