# Supplementary material for: Behavioural rhythms of two amphipod species Marinogammarus marinus and Gammarus pulex under increasing levels of light at night
Source: PLoS One. 2025 Aug 7;20(8):e0329449. doi: 10.1371/journal.pone.0329449 (PMC12331069; doi:10.1371/journal.pone.0329449)
Supplement: Attachment — Submitted filename: Response to Reviewers.docx [file pone.0329449.s006.docx]

Behavioural rhythms of two gammarid species Echinogammarus marinus and Gammarus pulex under increasing levels of light at night

Response to Reviewers

**We would like to thank the Reviewer and Editor for taking the time to address our manuscript. In making the suggested additions and changes, we believe that the paper has been significantly strengthened. We have provided our responses (in bold) beneath each comment (in italics), with line numbers referring to highlighted text in the Revised Manuscript with Tracked Changes. While we have double-checked that the line numbers are correct, there may still be discrepancies due to the tracked changes.**

**One important note, the name of *Echinogammarus marinus* was recently changed back to *Marinogammarus marinus.* This has been amended throughout the manuscript.**

*Journal Requirements:*

*When submitting your revision, we need you to address these additional requirements.*

*1. Please ensure that your manuscript meets PLOS ONE's style requirements, including those for file naming. The PLOS ONE style templates can be found at*

[*https://journals.plos.org/plosone/s/file?id=wjVg/PLOSOne_formatting_sample_main_body.pdf*](https://journals.plos.org/plosone/s/file?id=wjVg/PLOSOne_formatting_sample_main_body.pdf)

*and*

[*https://journals.plos.org/plosone/s/file?id=ba62/PLOSOne_formatting_sample_title_authors_affiliations.pdf*](https://journals.plos.org/plosone/s/file?id=ba62/PLOSOne_formatting_sample_title_authors_affiliations.pdf)

**We have reviewed the style requirements and made the appropriate changes to the author affiliations, reference formatting, and supplemental information.**

*2. In your Methods section, please provide additional information regarding the permits you obtained for the work. Please ensure you have included the full name of the authority that approved the field site access and, if no permits were required, a brief statement explaining why.*

**No permits were required for this work as the sites are open to the public and neither animal species falls under the requirements for ethics approval. This information has now been added to the Methods section (L123-125).**

*3. Thank you for stating the following financial disclosure:*

*“Natural Environment Research Council (grant number NE/S007210/1) Research England’s Expanding Excellence in England (E3) Fund”*

*Please state what role the funders took in the study. If the funders had no role, please state: "The funders had no role in study design, data collection and analysis, decision to publish, or preparation of the manuscript."*

*If this statement is not correct you must amend it as needed.*

*Please include this amended Role of Funder statement in your cover letter; we will change the online submission form on your behalf.*

**The funders had no role. This statement has now been added to the cover letter.**

*4. Thank you for stating the following in the Acknowledgments Section of your manuscript:*

*“This work was supported by the INSPIRE Doctoral Training Programme and the Natural Environment Research Council (grant number NE/S007210/1). Thanks to the Invertebrate Facility of the School of Biological Sciences at the University of Southampton for providing space and equipment for the experiments that were conducted in this study. SCR was partially funded by Research England’s Expanding Excellence in England (E3) Fund.”*

*We note that you have provided funding information that is not currently declared in your Funding Statement. However, funding information should not appear in the Acknowledgments section or other areas of your manuscript. We will only publish funding information present in the Funding Statement section of the online submission form.*

*Please remove any funding-related text from the manuscript and let us know how you would like to update your Funding Statement. Currently, your Funding Statement reads as follows:*

*“**Natural Environment Research Council (grant number NE/S007210/1) Research England’s Expanding Excellence in England (E3) Fund”*

*Please include your amended statements within your cover letter; we will change the online submission form on your behalf.*

**All funding information has now been removed from the text of the manuscript.**

*5. Thank you for stating in your Funding Statement:*

*“SCR was partially funded by Research England’s Expanding Excellence in England (E3) Fund.”*

*Please provide an amended statement that declares *all* the funding or sources of support (whether external or internal to your organization) received during this study, as detailed online in our guide for authors at http://journals.plos.org/plosone/s/submit-now. Please also include the statement “There was no additional external funding received for this study.” in your updated Funding Statement.*

*Please include your amended Funding Statement within your cover letter. We will change the online submission form on your behalf.*

**The Funding Statement has now been updated and this section removed as this grant did not directly fund this work in any way.**

*6. Please note that in order to use the direct billing option the corresponding author must be affiliated with the chosen institute. Please either amend your manuscript to change the affiliation or corresponding author, or email us at plosone@plos.org with a request to remove this option.*

**The corresponding author has not changed.**

*7. Please include captions for your Supporting Information files at the end of your manuscript, and update any in-text citations to match accordingly. Please see our Supporting Information guidelines for more information:* [*http://journals.plos.org/plosone/s/supporting-information*](http://journals.plos.org/plosone/s/supporting-information)*.*

**Captions for the Supporting information files have now been added to the end of the manuscript.**

*Reviewer #1: With respect
The study was re-examined statistically and content.
To further improve and improve the quality of study, do the following instructions.
Upon reviewing the manuscript titled "Behavioural rhythms of two gammarid species Echinogammarus marinus and Gammarus pulex under increasing levels of light at night", the following potential flaws were identified:
While the introduction provides a strong rationale, it lacks specific details about how the findings of this study advance the existing body of literature on artificial light at night (ALAN). Adding a clearer gap analysis could improve its positioning.*

**We have now included a more explicit gap analysis to the introduction (L91-94; L101-108) that highlights the limited number of studies conducted with these species and the fact that light pollution will directly interact with other known behavioural-altering stressors (such as parasitism and pharmaceutical contamination). It’s therefore necessary to get a baseline understanding of how light at night alters their behaviour so that future work can determine what the compounding effects of all these stressors might be.**

*The introduction predominantly focuses on the negative effects of ALAN, with little acknowledgment of any adaptive or neutral responses by species to light pollution. This creates a slightly biased narrative.*

**Thank you for pointing out this oversight. You are right that not all the impacts of ALAN have been detrimental for individual species. We have amended the text to be more neutral (L72-75).**
 *The use of constant darkness (DD) as a control raises questions.*

**To clarify, constant darkness was not used as a control. We did not adjust the data against DD for normalisation and we did not make comparisons against DD for a baseline. Both DD and LL were used to examine free-running rhythms as is common in studies related to the circadian clock as they represent the two extremes of the light regime scale (Stoleru et al., 2005; Bachleitner et al., 2007; Teschke et al., 2011; Zhang et al., 2013). As light is considered the primary environmental cue for the circadian clock, using a constant dark regime allows us to see how the clock runs without that input. Since studies of this nature have never been conducted with these two species, we felt it was important to include both DD and LL to understand their free-running rhythms. This has been included in the methods (L168-170).**

*The mortality observed in DD might have been influenced by oxygen deprivation, which was not controlled or monitored. Including oxygen level data for all conditions would strengthen the experimental rigor.*

**Overall mortality was low throughout the assays, including in the DD treatment for *G. pulex.* The water used in all assays was aerated for at least 24 hours to ensure high oxygen levels prior to being added to the vials at the beginning of every treatment, which proved sufficient for all other treatments. Amphipods have previously been shown to be quite tolerant of low-oxygen environments (Culver & Poulson, 1971; Agnew & Taylor, 1985; Maltby, 1995) so the high mortality may have been unrelated to oxygen deprivation. We have included this context in our Discussion as well (L372-377) but likewise suggest monitoring oxygen levels as a precaution*.* The animals that did not survive or were insufficiently active were excluded from the subsequent analyses so the results presented in the manuscript reflect the behaviour of live, motile animals.** **Finally, a clear impact of oxygen depletion on the ability to express normal activity patterns would likely result in a progressive change in the diel activity patterns towards the end of the experiment. Our data do not appear to show such effects in a specific manner for the DD condition.**
 *The study uses an equal number of males and females, but sex-specific behaviors were pooled for many analyses. This could obscure potentially important differences in how males and females respond to ALAN.*

**We also wanted to ensure there were no significant differences in the responses of males and females, which is why we directly compared their behaviour. Fortunately, we found only 5 instances (out of a possible 64) of significant differences across all treatments and measured behaviours for both species. This was, however, somewhat expected as none of the behaviours we examined would be considered sex-specific. For transparency, these differences have all been highlighted in the Results and Supplemental material. Given how few differences we found, we felt it was appropriate to pool the data for the subsequent analyses to improve their statistical power. Overall, the pooled results give an accurate indication of the species responses to light at night.**
 *While the study mentions the impact of ALAN on tidal rhythms briefly, it does not experimentally test the interaction between tidal and circadian cycles. This limits the ecological applicability of the findings for intertidal species.*

**The behavioural rhythms of intertidal organisms can be modulated by many factors including light cycles, tidal patterns, and temperature. It is common practice when investigating rhythmic behaviour to isolate specific zeitgebers by maintaining constant conditions. Similar work on intertidal organisms have alternately eliminated light cycles and tidal cycles to remove their influence on behaviour (Zhang et al., 2013; Kwiatkowsky et al., 2023). Since the focus of this research was on light pollution, we opted to maintain a constant temperature and forgo tidal cycles so that we could isolate the role of light in determining behaviour. We believe that the results presented here provide useful insight into how ALAN disrupts these rhythms.**

**However, we agree that in future studies tidal entrainment and temperature cycles could be included to get a more well-rounded understanding of their interactions with ALAN and we make these recommendations in the discussion (L395-410).**
 *Statistical tests were performed appropriately, but the results could benefit from a more detailed explanation of effect sizes to demonstrate biological relevance beyond statistical significance.*

**Thank you for pointing out this oversight. We have amended the Discussion to include more information about the biological relevance of our results (L333-340; L391-392; L420-421).**  *The manuscript uses multiple post-hoc comparisons (e.g., Bonferroni correction). However, no justification for choosing this conservative method over others is provided. Some alternative corrections might allow more nuanced insights into the data.*

**We reconsidered our use of Bonferroni corrections because, as the reviewer points out, they are overly conservative for our purposes. We re-ran the analyses with Benjamini-Hochberg corrections instead, which led to only minor changes in the results. We have amended the Results and Discussion sections to reflect these changes (L257-266; L318-320).**
 *Figures, while informative, are dense and require clearer labeling to aid comprehension. For example, Figures 2A-H include multiple parameters that could confuse readers unfamiliar with rhythmic activity metrics.*

**We have amended the figure legend to include more information for those unfamiliar with rhythmic activity metrics. We were unclear what other labelling could be helpful but would appreciate any specific suggestions that increase the accessibility of the results.**
 *The results section references supplementary material (e.g., "Fig S1C"), which is crucial for interpreting key findings. These supplementary figures should be integrated more effectively or summarized in the main text.*

**Figs S1-2 were relegated to the supplementary material as we felt they did not provide any nuanced insights regarding the data. They merely corroborate what is already stated in the results.**

**Figs S3-4 were not included in the main text primarily due to their size and complexity. While they illustrate some of the nuanced differences between treatments, the information they provide is better summarised by Fig 2 in the main text.**

*While the discussion highlights key findings, it sometimes speculates without strong evidence (e.g., oxygen levels in vials, resilience of Gammarus pulex). These points should be either substantiated or rephrased to reflect their speculative nature.*

**We have amended the text to emphasise that these comments are purely speculative.**
 *There is limited focus on how findings can be applied to mitigate ALAN's ecological effects. Including suggestions for practical conservation measures or further research directions would enhance the impact.*

**We have amended the Conclusions to include suggestions for how results such as ours can be integrated into policy changes that limit the impact of ALAN in natural ecosystems (L460-466).**
 *No discussion is provided on how ALAN exposure in controlled laboratory settings reflects real-world ecological conditions. This is particularly relevant given the mention of skyglow and natural light scattering in the introduction.*

**We have now acknowledged in the Conclusions that the results from our lab-based experiments would be further strengthened by complementary field-based studies in afflicted areas (L458-459).**
 *The manuscript generally maintains a high standard of academic language. However, some sentences, particularly in the discussion, are overly long and complex, potentially hindering readability.*

**After re-reading the manuscript, we have edited longer sentences for clarity.**
 *You can benefit from and cite the following studies to improve parts of the text:
- Knockdown resistance (kdr) associated organochlorine resistance in mosquito-borne diseases (Culex quinquefasciatus): Systematic study of reviews and meta-analysis
- Knockdown Resistance (kdr) Associated Organochlorine Resistance in Mosquito-Borne Diseases (Anopheles subpictus): Systematic Review Study
- Knockdown resistance (kdr) Associated organochlorine Resistance in mosquito-borne diseases (Culex pipiens): Systematic study of reviews and meta-analysis*

**Thank you for the recommendations. These analyses are insightful in their own right. We did struggle, however, to find an appropriate section in this manuscript in which to cite them. We may, instead, cite them in another upcoming paper associated with chemical control of insect pests.**
