# Supplementary material for: Behavioural rhythms of two amphipod species Marinogammarus marinus and Gammarus pulex under increasing levels of light at night
Source: PLoS One. 2025 Aug 7;20(8):e0329449. doi: 10.1371/journal.pone.0329449 (PMC12331069; doi:10.1371/journal.pone.0329449)
Supplement: Attachment — Submitted filename: Response_to_Reviewers_auresp_2.docx [file pone.0329449.s008.docx]

Behavioural rhythms of two gammarid species *Marinogammarus marinus* and *Gammarus pulex* under increasing levels of light at night

Response to Reviewers

**We would like to thank the Reviewer and Editor for taking the time to address our manuscript. The reviewer made some very insightful suggestions that have significantly strengthened our paper. We have provided our responses (in bold) beneath each comment (in italics), with line numbers referring to highlighted text in the Revised Manuscript with Tracked Changes. While we have double-checked that the line numbers are correct, there may still be discrepancies due to the tracked changes.**

*6. Review Comments to the Author

Please use the space provided to explain your answers to the questions above. You may also include additional comments for the author, including concerns about dual publication, research ethics, or publication ethics. (Please upload your review as an attachment if it exceeds 20,000 characters)*

*Reviewer #2: The present study deals with the alterations in the swimming behaviour of two gammarid species caused by ALAN. To address this issue, the authors acclimated and exposed the species Marinogammarus marinus and Gammarus locusta to increasing levels of artificial light, including light controls. The authors measured different behavioural parameters of both species, such as number of active individuals, period length of rhythmic or nocturnality, considering sex-specific responses. The topic of the study is manuscript is very interesting and necessary given the current illumination to which many shallow aquatic ecosystems are exposed. Furthermore, the manuscript is well-written, the experimental design is clear and robust, and the results are novelty, helping to fill a gap in the knowledge of the effects of light pollution on the organisms inhabiting the water column. Some important issues, mostly related to the experimental design, have been clarify in response to previous reviewers. There are, however, some points that must be addressed, for example, the quality of the introduction and also the method used by the authors to identify the species. I have also included some comments, suggestions and slight corrections below. For these reasons, I recommend the manuscript for publication pending these minor revisions.*

**Thank you. We greatly appreciate your feedback and suggestions.**  *Comments

Abstract line 37. The contracted form of a species cannot appear in the beginning of a sentence. Thus, “G. pulex” must be changed by “Gammarus pulex”. The same applies to “M. marinus” in the next sentence.*

**Thank you for pointing this out, we weren’t aware of this. This has been amended throughout the text.**

*Abstract line 41. Replace “-“ with “–“ to use the same one than in line 35.* **Amended**

*Introduction line 50. Replace “(4)” with “[4]”. In any case, I think that the paper that claimed the 2.2% increase is Kyba et al., 2017 (Artificially lit surface of Earth at night increasing in radiance and extent) instead of Davies et al., 2013.* **Thank you, that was likely an error with the reference manager. This has now been amended.**

*Introduction line 53. I believe that this increase of illuminance must be considered as artificial rather than “natural light”.* **Amended**

*Introduction line 75. Despite most of the mechanisms that cause the cascading effects are mostly unknown, I recommend going deeper into this point given the relevance in the structure of the community. Here I suggest some references dealing with these effects:
⎯ Bennie, J., Davies, T.W., Cruse, D., Inger, R., Gaston, K.J., 2015. Cascading effects of artificial light at night: resource-mediated control of herbivores in a grassland ecosystem. Philos. Trans. R. Soc., B 370 (1667), 20140131.* [*https://doi.org/*](https://doi.org/) *10.1098/rstb.2014.0131.
⎯ Bennie, J., Davies, T.W., Cruse, D., Inger, R., Gaston, K.J., 2018. Artificial light at night causes top-down and bottom-up trophic effects on invertebrate populations. J. Appl. Ecol. 55, 2698–2706.* [*https://doi.org/10.1111/1365-2664.13240*](https://doi.org/10.1111/1365-2664.13240)*.
⎯ Sanders, D., Gaston, K.J., 2018. How ecological communities respond to artificial light at night. J. Exp. Zool. Part A: Ecological and Integrative Physiology 329 (8–9), 394–400.* [*https://doi.org/10.1002/jez.215*](https://doi.org/10.1002/jez.215)*.*

**We have now expanded on this point in the introduction (L76-79)**

*Introduction line 75: This sentence seems to reduce all the ALAN effects to phototaxis, but in the next sentence there are included some examples that are not necessary depending on movement alterations (e.g. disruption in mating could be caused by ALAN alterations in bioluminescence signals). Furthermore, movement alterations can also include disorientation, as Gaston and Bennie (2014) considered in their classification of the different organisms’ response to ALAN (i.e. light distracted species, light-exploiter species and light-repelled species). Therefore, I strongly recommend reformulating this statement to consider a wider spectrum of responses, or perhaps just replacing “regardless of whether an animal is positively or negative phototactic” to “regardless of whether an animal response to ALAN”.
⎯ Gaston, K.J. & Bennie, J. (2014) Demographic effects of artificial nighttime lighting on animal populations. Environmental Reviews, 22(4), 323–330.* [*https://doi.org/10.1139/er-2014-0005*](https://doi.org/10.1139/er-2014-0005)*.*

**We did not intend to imply all ALAN effects came down to phototaxis so we have reworded this sentence to be more broad (L70-73).**

*Introduction line 78: In line 72 the authors mentioned the potential effects of ALAN in the community through cascading effects in some species. After that, the authors explained the potential alterations that ALAN can cause in the species, returning to the effects on the community structures and dynamics in the next sentence. I think that it would be clearer if the sentence “While some species remain unaffected…” were between “Regardless of whether an animal…” and “By providing more favourable conditions…”, but it is just a suggestion.*

**Thank you for the suggestion, we agree that does sound clearer.**

*Introduction line 87. Synonymized names cannot be placed before authorship. Please, replace it with “Marinogammarus marinus (Leach, 1816) (formerly Echinogammarus marinus)”.* **Amended**

*Introduction line 90. Is it possible to include some references to support their ecological role?* **Amended**

*Introduction line 91. There is a study dealing with the swimming behaviour of Gammarus pulex. Although it does not include an ALAN-approach, they found different behavioural patterns between dark and light conditions, in case the authors would like to take it into consideration:
⎯ Van der Berg, S.J.P., Rodríguez-Sánchez, P., Zhao, J., Olusoiji, O.D., Peeters, E.T.H.M., Schuijt, L. (2021). Among-individual variation in the swimming behaviour of the amphipod Gammarus pulex under dark and light condition. Science of the Total Environment, 782, 162177.* [*http://dx.doi.org/10.1016/j.scitotenv.2023.162177*](http://dx.doi.org/10.1016/j.scitotenv.2023.162177)

**We had not come across this paper before. While it doesn’t seem to fall under the category of ALAN research, it would be relevant to our Discussion, particularly on behavioural differences between the sexes.**

*Introduction line 95. Please, include some references to support the statement about the role of light in these species.*

**We’ve included an additional reference but the sentences following that statement and the references therein go into more specific detail regarding the role of light in these species.**

*Introduction line 97. Change “…exposed to light at night” by “…exposed to ALAN”.* **Amended**

*Introduction line 99. Change “…proliferation of artificial light” by “proliferation of ALAN.”* **Amended**

*Introduction line 101. I think that this should be in plural since the authors have explained the potential effect of ALAN in these species using several papers.* **Amended**

*Introduction line 108. After reading the whole Introduction, I think that this section would benefit from including additional examples related to the ecological impacts of ALAN, as I claimed in my comments above. There are several examples of specific and/or ecological alterations caused by ALAN in aquatic ecosystems that the authors could use to provide a greater context. Although it is true that the impact of ALAN in biological clocks still remains unexplored (which supports the relevance of the present study), a deeper understanding of how ALAN disrupts the ecosystem is mandatory for Introduction. I suggest this study by Sanders and Gaston (2018) but, as I said, there are examples of alterations in particular species, interactions or habitats that the authors could use.
⎯ Sanders, D., Gaston, K.J., 2018. How ecological communities respond to artificial light at night. J. Exp. Zool. Part A: Ecological and Integrative Physiology 329 (8–9), 394–400.* [*https://doi.org/10.1002/jez.215*](https://doi.org/10.1002/jez.215)

**We have now included more examples of the ecological impacts of ALAN in the introduction (L76-79).**

*Methods line 111. This is the first time that the species in mentioned in this section, so it should be appeared as “Marinogammarus marinus”, but without the authorship.* **Amended**

*Methods line 113. Replace it with “Gammarus pulex”.* **Amended**

*Methods line 125. There are several species of this family in the British coasts (e.g. Marinogammarus obtusatus, Gammarus chevreuxi, Gammarus locusta, etc.), some of them coexisting with M. marinus. I believe that it is very important that the authors indicate in this subsection if they have used some key or the characters to identify these species and also if the differentiation from others in the same habitat was conducted in situ or in the laboratory.*

**While it is true that those species often coexist, we collected our specimens from Lock Lake in Langstone Harbour, an area that has been extensively studied for decades due to its proximity to the University of Portsmouth’s Institute of Marine Sciences (IMS). In addition to visual identifications, the wider team at IMS has done substantial transcriptomic and genomic work on this population and has only ever found *M. marinus* based on CO1 sequences. For those reasons, we are very confident that the animals we collected were only *M. marinus*. We have added a line to address this (Methods L118-120).**

**-Cogne, Y., Degli-Esposti, D., Pible, O. *et al.* 2019. *De novo* transcriptomes of 14 gammarid individuals for proteogenomic analysis of seven taxonomic groups. *Scientific Data* 6, 184.** [**https://doi.org/10.1038/s41597-019-0192-5**](https://doi.org/10.1038/s41597-019-0192-5)

**-Short, S., Yang, G., Guler, Y., Etxabe, A.G., Kille, P., and Ford, A.T. Crustacean Intersexuality Is Feminization without Demasculinization: Implications for Environmental Toxicology. *Environmental Science & Technology* 2014 *48* (22), 13520-13529.** [**https://doi.org/10.1021/es5050503**](https://doi.org/10.1021/es5050503)

*Methods line 129. Replace it with “Fucus vesiculosus”.* **Amended**

*Methods line 129. Since M. marinus is an intertidal species, were some pebbles or rocks from its habitat added to the bottom of the tank or just the algae? Although algae provide them shelter, the absence of their natural elements could stress them.*

**The habitat in Langstone Harbour is quite sandy/silty so in earlier trials of the experiment, we did include aquarium sand in the vials. We found that the animals did not react well to it and though the animals survived, there was almost no activity detected. Since they tend to cling to *F. vesiculosus* in the wild, we decided it was best to only include the seaweed.**

*Methods line 140. Males and females are very similar in genera Marinogammarus and Gammarus with few exceptions that usually requires binocular stereoscope (e.g. setation in first antenna or shape of second gnathopod). Were both sexes differentiate based only on the body size or did the authors use sexual characters? Please clarify the method to differentiate sex.*

**Individuals were sexed based on their gnathopod size and the uropod setae. This has now been included in the Methods section (L152-154).**

*Methods line 187. The authors use indistinguishably the terms gammarids and amphipods. Although in this context they are quite similar, they are not, so I recommend choosing one throughout the manuscript when referring to Marinogammarus and Gammarus.*

**We have changed all terms to amphipod throughout the manuscript.**

*Methods line 201. Was this high mortality caused directly by the effects of the darkness in the animal or indirectly by the death of algae?*

**We believe it was caused indirectly by the death of the algae but we cannot conclusively link the mortality to any one cause.**

*Methods line 201. The authors explain that they discarded one channel since there were G. pulex in the vial of M. marinus. As I said above, I believe that it is necessary for the authors to include the characters to differentiate both species.*

**To clarify, *G. pulex* was not in the vial of *M. marinus*. There were two *G. pulex* in the same vial. The two species were collected and housed separately. Their assays were also conducted in separate monitors, usually on separate days. It was therefore unlikely that the two species would be misidentified.**

*Methods line 203. Replace with “Marinogammarus marinus”.* **Amended**

*Results line 223. Replace with “Marinogammarus marinus”.* **Amended**

*Results line 225. In figure 1, 2 and S1 the species Marinogammarus marinus still appears as E. marinus. Please, replace them with “M. marinus”. Furthermore, in the caption of all the figures should appear the complete name of the species, not the contracted form.* **Amended**

*Results line 231. Replace with “Gammarus pulex”.* **Amended**

*Discussion line 313. Replace with “Marinogammarus marinus”.* **Amended**

*Discussion line 314. Replace with “Gammarus pulex”.* **Amended**

*Discussion line 316. Replace with “Marinogammarus marinus”.* **Amended**

*Discussion line 322. In Van der Berg et al (2021) they discussed the observed differences in the swimming behavior of G. pulex according to sex with previous results in the literature. I believe that this point of the discussion would benefit from the proposed paper since there are few references to explain the lower activity in females.
⎯ Van der Berg, S.J.P., Rodríguez-Sánchez, P., Zhao, J., Olusoiji, O.D., Peeters, E.T.H.M., Schuijt, L. (2021). Among-individual variation in the swimming behaviour of the amphipod Gammarus pulex under dark and light condition. Science of the Total Environment, 782, 162177.* [*http://dx.doi.org/10.1016/j.scitotenv.2023.162177*](http://dx.doi.org/10.1016/j.scitotenv.2023.162177)

**Thank you for this paper suggestion. We have incorporated some of their results in our Discussion (L317-319).**

*Discussion line 331. Replace with “Fucus vesiculosus”.* **Amended**

*Discussion line 329. Since feeding and avoiding predation are probably the main contributions to the different light responses, there may be other factors that regulate their swimming behaviour and thus, the effect of ALAN. For example, Rygg (1972) studied the effect of light in the swimming behaviour of different gammarids from genera Marinogammarus and Gammarus and he observed differences during the ontogeny in some species. Therefore, if the population dynamic is not the same between both species this may cause slight differences. Of course, it is not possible to cover all the intraspecific variables in the experiment (indeed I consider robust the differentiation between males and females carried out in the present study), but I think that this paragraph should indicate that there may more factors behind the differences between both species.
⎯ Rygg, B. (1972). Factors controlling the habitat selection of Gammarus duebeni Lillj. (Crustacea, Amphipoda) in the Baltic. Annales Zoologici Fennici, 9(3), 172–183.*

**Thank you for pointing that out. We agree that there could be other factors we haven’t considered. We have reworded this sentence to acknowledge this (L324-327).**

*Discussion line 341. Replace with “Gammarus pulex”.* **Amended**

*Discussion line 363. Replace with “Marinogammarus marinus”.* **Amended**

*Discussion line 363. I agree with the authors, the low survival rate could be due to other factors unrelated to light. In fact, in some gammarids species there is cannibalism from the biggest males, even when algae are present. I do not know if the authors observed any sign of cannibalism in the dead specimens, but if so, it might be interesting to include this possibility in the potential causes.*

**It is true that some gammarids exhibit cannibalism and this was observed in the holding tanks. During the assays, however, the animals were assigned to individual vials so cannibalism would not explain those dead specimens.**

*Discussion line 378. Replace with “ALAN”.* **Amended**

*Discussion line 385. Include the authorship: “Orchestoidea tuberculata Nicolet, 1849”.* **Amended**

*Discussion line 405. Include the authorship: “Parhyale hawaiensis (Dana, 1853)”. Additionally, this species is not considered as talitrid but hyalid.* **Amended**

*Discussion line 435. Discussion would benefit from including potential alterations in the community caused by the observed effects of ALAN in both species. Indeed, in Introduction section the authors rightly mentioned that light pollution has the potential to disrupt the communities through cascading effects on single species. However, little has been discussed about the ecological consequences of the suppression of swimming behaviour. Although this may be merely speculative, there is literature about the ecological role of amphipods (including gammarids) in benthic habitats, so I strongly recommend including a few lines of the potential consequences in the community or the ecosystem when they are affected.*

**We have added a few more sentences to highlight the ecological role of each species and that changes in their behaviour could lead to cascading impacts to the community (L335-337; L359-364; L423-424).**

*Discussion line 443. Although this paper dealt with the combined effects of ALAN and ocean warming on productivity parameters (in copepods) rather than in circadian clocks, I think that the incorporation of this study may help with this part of the Discussion:
⎯ Nguyen, T.T., Le, M.-H., Doan, N.X., Pham, H.Q., Vu, M.T.T., Dinh, K.V., 2020. Artificial light pollution increases the sensitivity of tropical zooplankton to extreme warming. Environ. Technol. Innov. 20, 101179.* [*https://doi.org/10.1016/j.eti.2020.101179*](https://doi.org/10.1016/j.eti.2020.101179)

**Thank you. This paper is still highly relevant to the broader point we were trying to make. We have reworded this sentence to include parameters beyond circadian clocks explicitly (L442-445).**

*Conclusions line 455. This conclusion is focused on the generalize problematic of light pollution, rather than on the obtained results. Indeed, the only mention to the novel results is the following: “This study illustrates that artificial light at night can have strong but disparate effects on behavioural rhythms across closely related species”. The present study has revealed very interesting points about the swimming response of two similar species, including sexual differences, therefore, I do not understand why all these results are missing. Conclusion should be modified to include the novelties of the present work, keeping of course the issue of light pollution and the need to study a wider number of species in order to understand the true magnitude of this hazard.*

**Initially, we aimed to use the Conclusions section to move beyond our results to the broader future and applications of ALAN research. However, we now agree that it’s best to highlight our results to further emphasise the risks of ALAN exposure. We have more explicitly outlined our results (L461-465).**
